# Advantages and applications of CAR-expressing natural killer cells

**DOI:** 10.3389/fphar.2015.00021

**Published:** 2015-02-12

**Authors:** Wolfgang Glienke, Ruth Esser, Christoph Priesner, Julia D. Suerth, Axel Schambach, Winfried S. Wels, Manuel Grez, Stephan Kloess, Lubomir Arseniev, Ulrike Koehl

**Affiliations:** ^1^Institute of Cellular Therapeutics Integrated Research and Treatment Center Transplantation, Hannover Medical SchoolHannover, Germany; ^2^Institute of Experimental Hematology, Hannover Medical SchoolHannover, Germany; ^3^Georg-Speyer-Haus, Institute for Tumor Biology and Experimental TherapyFrankfurt am Main, Germany

**Keywords:** CAR, suicide genes, NK cells, T cells

## Abstract

In contrast to donor T cells, natural killer (NK) cells are known to mediate anti-cancer effects without the risk of inducing graft-versus-host disease (GvHD). In order to improve cytotoxicity against resistant cancer cells, auspicious efforts have been made with chimeric antigen receptor (CAR) expressing T- and NK cells. These CAR-modified cells express antigen receptors against tumor-associated surface antigens, thus redirecting the effector cells and enhancing tumor-specific immunosurveillance. However, many cancer antigens are also expressed on healthy tissues, potentially leading to off tumor/on target toxicity by CAR-engineered cells. In order to control such potentially severe side effects, the insertion of suicide genes into CAR-modified effectors can provide a means for efficient depletion of these cells. While CAR-expressing T cells have entered successfully clinical trials, experience with CAR-engineered NK cells is mainly restricted to pre-clinical investigations and predominantly to NK cell lines. In this review we summarize the data on CAR expressing NK cells focusing on the possible advantage using these short-lived effector cells and discuss the necessity of suicide switches. Furthermore, we address the compliance of such modified NK cells with regulatory requirements as a new field in cellular immunotherapy.

## INTRODUCTION

Cell-based therapies are becoming more and more important for the treatment of disease progression in cancer, severe infection, or GvHD occurring after stem cell (SCT) or organ transplantation. Beside hematopoietic stem cells, dendritic cells, mesenchymal stromal cells, unselected T lymphocytes, and antigen-specific or regulatory T cells, alloreactive NK cells are currently getting into the focus of interest as suitable and powerful effector cells for cellular therapy of cancer (Velardi, 2012). NK cells are defined as CD56+ and CD3- cells and are subdivided into cytotoxic CD56^dim^CD16^+^ and immunoregulatory CD56^bright^CD16^-^ cells. NK cells are of great clinical interest because they contribute to the graft-versus-leukemia/tumor (GvL/GvT) effect but are not responsible for the GvHD. However, the strong cytotoxicity of NK cells can be hampered by various tumor immune escape mechanism (Raffaghello et al., 2004; Holdenrieder et al., 2007; Kloess et al., 2010). In order to improve cytotoxic activity, effector cells can be efficiently and specifically redirected by recombinant chimeric antigen receptors (CARs), which consist of a single-chain variable fragment (scFv; ectodomain) linked to intracellular signaling domains (endodomain). The scFv binds to a defined target antigen on, i.e., cancer cells and triggers effector cell activation upon target engagement.

Extensive pre-clinical studies over the last decades have led to successful clinical phase I/II studies with CAR expressing T cells in hematological malignancies, including lymphoma, chronic lymphocytic leukemia (CLL), and acute lymphoblastic leukemia (ALL; Brentjens et al., 2010; Porter et al., 2011; Kochenderfer et al., 2012, 2013; Grupp et al., 2013; Maude et al., 2014). Especially CD19 CAR T cells have induced long-term remissions in patients with B cell malignancies (Grupp et al., 2013). Currently, a broad range of different cancer target antigens is under clinical investigation in several clinical CAR T cell trials such as CD20, CD30, CD138, c-Met, EGFRvIII, FAB, GD2, HER2, WT1, PSMA, NY-ESO1, and others as reviewed in Corrigan-Curay et al. (2014) and Maus et al. (2014) and even more target antigens are under pre-clinical development (Kenderian et al., 2014; Leuci et al., 2014). In contrast to numerous pre-clinical and clinical trials in the context of CAR-modified T cells, little is known on CAR-engineered NK cells. Therefore the present review focuses on engineered NK cells from pre-clinic to clinic and addresses the question of the necessity of suicide switches to improve the safety of CAR-expressing NK cells.

## NK CELL SOURCES

Natural killer cells can be generated from different sources such as peripheral blood (PB), unstimulated leukapheresis products (PBSC), umbilical cord blood (UCB), bone marrow (BM), human embryonic stem cells (hESCs) or induced pluripotent stem cells (iPCSs; Woll et al., 2009; Chouaib et al., 2014). While the generation of NK cells from hESCs or iPCS has been largely experimental to date, the *ex vivo* expansion and NK differentiation of UCB-derived CD34^+^ cells has been successfully translated to the clinics (Spanholtz et al., 2011). An ongoing phase I clinical trial uses NK cells produced from CD34^+^ hematopoietic precursors to treat acute myeloid leukemia (AML) in elderly patients (CCMO nr. NL31699 and Dutch Trial Register nr. 2818). In the last decade effective methods for clinical grade purification and expansion of donor NK cells from PB and PBSC have been established successfully in order to obtain large numbers of NK cells (Iyengar et al., 2003; Koehl et al., 2005, 2013; Miller et al., 2005; Sutlu et al., 2010; Leung, 2011; Leung et al., 2014). In this respect, feasibility and safety of NK cell therapies has been shown in several phase I/II trials performing both the adoptive transfer of donor NK cells without transplantation (Miller et al., 2005) or donor-derived allogeneic NK cells post-SCT (Koehl et al., 2004; Stern et al., 2013; Leung et al., 2014). Depending on the source and the protocol, more immature, such as polyfunctional CD56^dim^KIR^+^CD62L^+^ or mature terminal effector CD56^dim^KIR^+^NKG2A^-^CD62L^-^ NK cells are available for the use in clinical studies (Luetke-Eversloh et al., 2013).

Next to primary human NK cells, cell lines can also be useful for allogeneic NK cell therapy. Several human NK cell lines have been established, i.e., NK-92, HANK-1, KHYG-1, NK-YS, NKG, YT, YTS, NKL reviewed in Kornbluth et al. (1985) and NK3.3 Cheng et al. (2012). Among them, the NK-92, KHYG-1, NKL, and NKG cell lines exert well-documented antitumor activities (Yagita et al., 2000). Beyond these pre-clinical investigations, NK-92 has also entered clinical trials successfully (Tonn et al., 2013).

## CAR EXPRESSING NK CELLS

*Ex vivo* expanded primary human NK cells produce a different spectrum of cytokines compared to T cells including γ-Interferon, IL-3 and the granulocyte macrophage colony stimulating factor (GM-CSF; Huenecke et al., 2010; Klingemann, 2014). CAR-modified NK cells can represent a complementary therapeutic option to CAR-expressing T cells. To date, pre-clinical data have been reported for CAR-modified primary human NK cells redirected against CD19, CD20, CD244, and HER2 as well as CAR-expressing NK-92 cells targeting a broader range of cancer antigens (**Table 1**).

**Table 1:** Pre-clinical trials using CAR-engineered primary human NK cells and Pre-clinical investigations of CAR-expressing NK-92 cells.

Literature	Target
**Pre-clinical investigations of CAR-expressing NK-92 cells**	
Boissel et al. (2009)	Chronic lymphocytic leukemia	CD19
Boissel et al. (2013)	Lymphoblastic leukemia	CD19;CD20
Chang et al. (2013)	Enhancement of NK cytotoxicity	NKG2D
Chu et al. (2014a)	Multiple Myeloma	CS1
Esser et al. (2012)	Neuroblastoma	GD2
Jiang et al. (2014)	Multiple Myeloma	CD138
Muller et al. (2008)	Lymphoma and leukemia	CD20
Sahm et al. (2012)	Breast carcinoma	EpCAM
Schonfeld et al. (2014)	Tumors of epithelial origin (breast carcinoma, pulmonary metastasis of renal cell carcinoma)	HER-2
Tassev et al. (2012)	EBV positive T cells	EBNA3C
Uherek et al. (2002)	Tumors of epithelial origin (breast carcinoma, ovarian carcinoma and epidermoid carcinoma cell lines)	HER-2 (ErbB2)
Zhang et al. (2013)	Melanoma	GPA7
**Pre-clinical trials using CAR-engineered primary human NK cells**	
Alsamah and Romia (2014)	HER-2 expressing cell lines	HER-2
Altvater et al. (2009)	Neuroblastoma	CD244
Chu et al. (2014b)	Burkitt lymphoma	CD20
Imai et al. (2005)	Leukemia	CD19
Kruschinski et al. (2008)	Ovarian cancer	HER-2
Li et al. (2010)	CD 19+ B-ALL derived OP-132 cell line	CD19
Shimasaki et al. (2012)	Leukemia	CD19
**Studies**
NCT 00995137	Genetically modified haploidentical natural killer cell infusions for B-lineage acute lymphoblastic leukemia	National Cancer Institute (NCI) David Shook, MD
NTC 01974479	Pilot study of redirected haploidentical natural killer cell infusions for B-lineage acute lymphoblastic leukemia	National University Health System, Singapore, Dario Campana

Several of the pre-clinical studies with CAR-expressing NK cells focus on anti-CD19 and anti-CD20 CARs targeting B cell malignancies (Imai et al., 2005; Muller et al., 2008; Boissel et al., 2009, 2013; Li et al., 2010; Shimasaki et al., 2012). Other pre-clinical studies have used the ganglioside GD2 as an antigen to target neuroblastoma (Altvater et al., 2009; Esser et al., 2012). An additional field for anti-GD2 CAR therapy could be opened by the finding that breast cancer stem cells are GD2 positive as well (Battula et al., 2012). HER2 may represent another attractive target for CAR-based immunotherapies if current safety concerns can be adequately addressed (Morgan et al., 2010). HER2 is overexpressed in 30–80% of human breast, ovarian, pancreatic, colon, gastric, lung, prostate carcinomas as well as melanomas, and correlates with a more aggressive disease progression (Uherek et al., 2002; Kruschinski et al., 2008; Schonfeld et al., 2014). A CAR targeting the pan-cancer antigen “Epithelial cell adhesion molecule” (EpCam) was successfully tested in NK-92 cells (Sahm et al., 2012). Further, CARs, that have been pre-clinically evaluated in the NK cell context, target GPA7 (Zhang et al., 2013), CD138 (Jiang et al., 2014), and CS1 (Chu et al., 2014a), which are present on multiple myeloma. Retroviral transduction of an NKG2D-DAP10-CD3ζ CAR that utilizes the extracellular domain of NKG2D for recognition of natural NKG2D ligands on the tumor cell surface, markedly increased NKG2D surface expression in NK cells, which became more cytotoxic against leukemia and solid tumor cell lines (Chang et al., 2013).

Importantly, also non-cancer targets can be eliminated by redirected NK cells. For example, an anti-EBNA3C CAR was used for the NK cell-mediated destruction of EBV positive T cells (Tassev et al., 2012) and an anti-CD4 CAR for the elimination of HIV-infected cells (Ni et al., 2014), overall demonstrating the huge potential of CAR-modified NK cells. To date, investigations about CAR expressing NK cells report on the overall NK cells and do not differ between specific NK cells subpopulations and maturation status of the NK cells. Mature donor NK cells purified from PB and activated with cytokines have a limited lifespan of a few days up to a few weeks (Brehm et al., 2011, 2014). Hence, allogeneic CAR-engineered NK cells are expected to be rejected or exhausted after destroying the cancer cells. In contrast, more immature NK cells derived from cord blood (Spanholtz et al., 2011) or from iPSC (Ni et al., 2014) have a longer lifespan which may allow extended antitumor activity, but also increases the risk of malignant transformation and other adverse effects, thus requiring improved safety strategies.

## VECTORS FOR TRANSDUCTION OF NK CELLS AND TRANSDUCTION EFFICACY

Stable gene transfer is required to enable sustained CAR expression in expanding and persisting effector cells (Sadelain et al., 2013). CAR endodomains are responsible for transmitting the activating signal within the lymphocytes. In first generation CARs, usually the cytoplasmic CD3ζ domain of the TCR complex was used as an endodomain (Irving et al., 1993). The potency of CARs can be increased by addition of one (second generation CAR) or more co-stimulatory domains (third generation CAR), derived from, e.g., CD28, OX-40, or 4-1BB. To increase the specificity of effector cells, also two separate chimeric proteins can be co-expressed, one representing a low-affinity activating CD3ζ receptor specific for a first tumor-associated antigen, the other receptor harboring a co-stimulatory domain fused to an antigen-binding domain targeting a second antigen. Only when both components are triggered, the effector cells will sufficiently be activated to achieve cytolytic activity (Park et al., 2011; Sadelain et al., 2013; Corrigan-Curay et al., 2014). In general, CAR affinity and functionality is determined by the interplay between the antigen-binding scFv (single chain variable fragment) antibody, hinge region, transmembrane region, and endodomain. Thus, CARs should be experimentally optimized for each target antigen and application.

Retro- or lentivirus-based vectors have mostly been used for CAR-engineered NK cells with various transduction efficacies. Imai et al. (2005) reported retrovirus-based transduction of a CD19-specific CAR construct with an efficacy of 43–93% in expanded primary NK cells. Another method of CAR transfection into NK cells is electroporation of plasmid DNA or mRNA (Li et al., 2010). In one study a comparison of mRNA transfection and lentivirus-based transduction of CD19 and CD20 CAR constructs showed a lower efficacy (<10%) in the electroporation approach in PB and cord blood NK cells, in comparison to a transduction efficacy for lentiviral vectors of 8–16% for PB and 12–73% for cord blood NK cells. Clinically relevant levels of mRNA transfection were obtained with the NK-92 cell line (Boissel et al., 2009). Likewise, Shimasaki et al. (2012). showed highly efficient expression of anti-CD19 chimeric receptors in either freshly purified (median, 40.3%) or expanded NK cells (median, 61.3%) after electroporation of the corresponding mRNA. During transduction of the NK cell, the CAR encoding vector is semi-randomly and stably incorporated into the host cell genome. This ensures long-lasting CAR expression, but may also lead to severe side effects, such as insertional mutagenesis and – in case of antigen healthy tissues – also persisting off-tumor/on-target activity. Numerous clinical studies have been performed with gamma-retro- and lentiviral vectors targeting hematopoietic stem and progenitor cells. While these approaches have clearly demonstrated efficacy, especially in inherited immunodeficiency disorders, leukemias due to insertional mutagenesis have been observed in a number of studies using gamma-retroviral vectors for the treatment of X-linked severe combined immunodeficiency (Hacein-Bey-Abina et al., 2008; Howe et al., 2008), chronic granulomatous disease (Stein et al., 2010), or the Wiskott–Aldrich syndrome (Braun et al., 2014). Improvements in vector architecture including the design of so-called SIN (self-inactivating) vectors have allowed second generation trials, which so far have shown a more favorable biosafety spectrum with no leukemia documented up to now (Aiuti et al., 2013; Biffi et al., 2013; Hacein-Bey-Abina et al., 2014). Nevertheless, clonal dominance, i.e., the strong prevalence of a few specific clones dominating hematopoiesis, has also been observed with second generation SIN vectors (Cavazzana-Calvo et al., 2010), arguing for careful vector design and/or additional inclusion of suicide switches even in these vectors.

In this respect, numerous clinical trials using LTR (long terminal repeat)-driven gamma-retroviral vectors for transduction of T cells have failed to show severe adverse events related to insertional mutagenesis reviewed in (Suerth et al., 2012) underlining the more favorable biosafety pattern of retroviral applications in mature lymphoid cells, such as T cells. Although these results are likely transferable to NK cells, a systematic biosafety analysis in NK cells with respect to vector architecture remains to be accomplished.

## SUICIDE SYSTEMS

Different safety concerns are associated with CAR-engineered effector cells, which include induction of (acute) GvHD as well as on-target/off-tumor effects, tumor lysis syndrome and cytotoxicity to normal tissues due to limited selectivity of the chosen target antigen (Ferrara et al., 2009; Stauss and Morris, 2013; Maus et al., 2014). To date, these observations are mainly restricted to CAR-expressing T cells, but are discussed for CAR-engineered NK cells as well, especially for immature NK cells.

Suicide gene therapy was first introduced in the context of human SCT performed in the 1990s using the herpes simplex thymidine kinase (HSV-TK)/Ganciclovir (GCV) suicide system (Bonini et al., 1997). Although its safety and efficacy was demonstrated, the HSV-TK system is severely limited by only being effective in proliferating cells. Due to its viral origin, HSV-TK is also immunogenic and can therefore cause the rejection of modified cells by the immune response of the host, although it has led to effective abrogation of GvHD in all clinical trials (Bonini et al., 2007). To overcome the limitations of the HSV-TK system, various alternative suicide gene systems have been developed. Previously we could demonstrate the B cell molecule CD20 as an effective suicide marker for genetically modified T cells (Vogler et al., 2010). CD20 expression on the cell surface allows elimination of the cells upon administration of rituximab. In contrast to HSV-TK, CD20 as a suicide gene may not be immunogenic due to its human origin, and elimination by rituximab is not affected by the cells’ proliferation status. Nevertheless, bio-distribution of the monoclonal antibody may be sub-optimal, and normal B cells are also depleted. Different apoptotic pathways have been employed as suicide systems, including the death receptor Fas and caspase 9 (CASP9; Fan et al., 1999; Di Stasi et al., 2011). Beside very low risk for immunogenicity, these suicide genes share the advantages of non-cell cycle dependency, full clinical compatibility and optimal bio-distribution, as CIDs are small molecules exquisitely designed for suicide purposes (Lipowska-Bhalla et al., 2012). A summary of the different suicide systems was published recently by Jones (Jones et al., 2014).

None of the aforementioned suicide systems has been intensively tested in NK cells. To date, suicide genes are primarily discussed in the context of long-living genetically modified effector cells. In contrast, mature allogeneic CAR-engineered NK cells are expected to induce anti-cancer effects and disappear after a few days. Therefore, the necessity of a suicide switch might not be given (Klingemann, 2014). However, in the context of more immature CAR expressing NK cells, suicide switches might be beneficial considering that UCB-derived NK cells or immature NK cells such as polyfunctional CD56^dim^KIR^+^CD62L^+^ cells have a lifespan up to months. Worth mentioning would also the finding, that IL15/4-1BBL activated NK cells could contribute to an acute GvHD caused by inflammation or other factors up-regulating activating ligand expression on non-malignant tissues (Shah et al., 2014).

## ENGINEERED NK CELLS: FROM PRECLINICAL STUDIES TO CLINIC APPLICATION

When compared to CAR T cells, experience with CAR-engineered NK cells is still limited. In contrast to a large number of clinical studies using CAR T cells for the treatment of various cancers, only two clinical studies employing CAR-expressing NK cells have been approved by the regulatory authorities and are open for patient acquisition. The longest open study is the pediatric study at St. Jude Children’s Research Hospital using haploidentical NK cells modified with anti-CD19 CARs for the treatment of B-lineage ALL (ClinicalTrials.gov. NCT00995137). In this study, the donor-derived NK cells are expanded by co-culture with the irradiated K562 cell line expressing membrane bound IL-15 and 41BB ligand (K562-mb15-41BBL). Another study for pediatric and adult patients with refractory ALL is the recently approved study at the National University Hospital in Singapore (ClinicalTrials.gov. NCT01974479). In this study, haploidentical NK cells will be activated by incubation with IL-2 and transduced with the same construct as employed in the St. Jude trial (Shimasaki and Campana, 2013). A number of scientific questions and regulatory hurdles have to be adressed before NK CAR cellular therapy can be extended to larger patient cohorts in phase III studies, such as manufacturing issues. Although powerful methods for isolation, expansion, and transduction have been described, the preparation of the cells is still cumbersome and currently restricted to highly specialized laboratories.

## MANUFACTURING OF REDIRECTED NK CELLS AND REGULATORY ISSUES IN EUROPE

According to their biological and pharmacological complexity CAR/suicide gene expressing NK cells will be classified as advanced therapy medicinal products (ATMP) in Europe and regulated either centralized or under the hospital exemption by the member states [Regulation (EC) No 1394/2007, Directive 2001/83/EC, and Regulation (EC) No 726/2004]. Although the primary therapeutic effect remains NK cell-specific the significant alteration of targeting by introduction of CAR will result in a gene therapy medicinal product (GTMP), whereas lifespan control by engineered suicidality only would result in a somatic cell therapy medicinal product (CTMP) [EMA/CAT/600280/2010 Rev.1, 20 June 2014].

**FIGURE 1:**
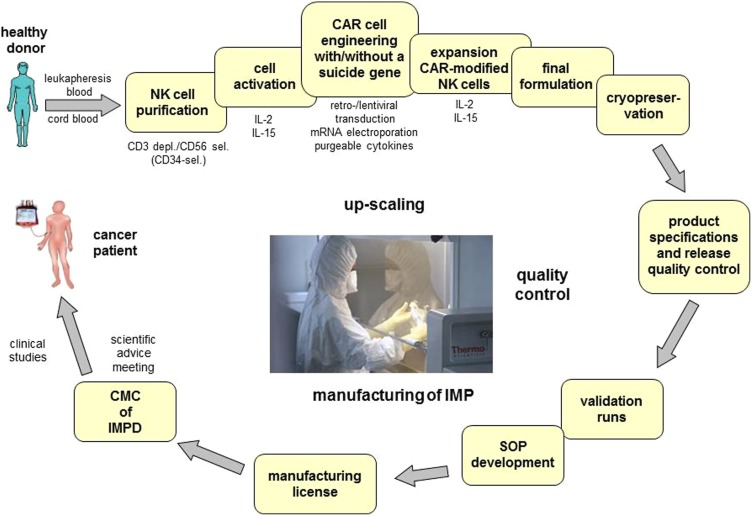
**GMP-conform manufacturing of chimeric antigen receptor expressing natural killer cells with/without suicide genes for safety improvement.** One major technical obstacle for the wide spread application of CAR expressing NK cells in cancer is the complexity of the GMP-conform manufacturing process of these CAR-ATMPs. The development of this process comprises not only the set-up of GMP-conform protocols for isolation, activation, engineering and expansion of the cells but also the final formulation, definition of product specifications and release quality control. In order to enter a clinical phase I/II trial the corresponding SOPs will be generated, validation runs will be performed and the application for manufacturing licenses has to be submitted. Due to the maturation status of the NK cells suicide genes might be necessary to improve safety in the use of CAR-engineered NK cells. CAR, chimeric antigen receptor; ATMP, advanced therapy medicinal products; depl., depletion; sel., selection; SOP, standard operation protocol; IMP, investigational medicinal product; IMPD, investigational medicinal product dossier; CMC, chemical manufacturing and control.

For successful clinical translation of gene modified NK cells, the preclinical and clinical development (**Figure 1**) will have to focus on the transduction efficacy, as well as on the safety and efficacy of the CAR and/ or the suicide constructs introduced. Therefore quality aspects related to CTMP and GTMP as defined in guidelines [CPMP/BWP/3088/99; EMEA/CHMP/410869/2006; *Ph. Eur. 0784: Ph. Eur. 5.14*] will apply to the identity, potency, and activity including the conditional suicidality, purity, and safety of vectors and genetically modified product. The establishment of correspondingly adequate in-process and quality controls as well as of process target values and product specifications will have to take into account the variability of the primary effector cell as the starting material (Schule et al., 2010).

## OUTLOOK

To date suicide gene-modified T cells and CAR expressing T cells have successfully entered clinical trials. For future clinical applications, a combination of both modifications might lead to an improved safety strategy. In contrast to gene-modified T cells, experiments with modified primary human NK cells are mainly restricted to the pre-clinical setting with promising results. Importantly, the first two clinical studies using CAR-expressing NK cells have started very recently. Due to the limited lifespan of mature CD56^dim^KIR^+^NKG2A^-^CD62L^-^NK cells, suicide genes might not be necessary, but this could be different for more immature CD56^dim^KIR^+^CD62L^+^ NK cells derived from cord blood or iPS cells. While theoretical risk-benefit considerations, only argue for the incorporation of suicide switches in the context of CAR-modified and/ or more immature NK cells, careful preclinical investigations are needed to provide a final answer in the future.

## Conflict of Interest Statement

The authors declare that the research was conducted in the absence of any commercial or financial relationships that could be construed as a potential conflict of interest.
